# Flavones as Quorum Sensing Inhibitors Identified by a Newly Optimized Screening Platform Using *Chromobacterium violaceum* as Reporter Bacteria

**DOI:** 10.3390/molecules21091211

**Published:** 2016-09-10

**Authors:** Malena E. Skogman, Sonja Kanerva, Suvi Manner, Pia M. Vuorela, Adyary Fallarero

**Affiliations:** 1 Pharmaceutical Design and Discovery Group (PharmDD), Division of Pharmaceutical Biosciences, Faculty of Pharmacy, Viikinkaari 5E, University of Helsinki, FI-00014 Helsinki, Finland; malena.skogman@helsinki.fi (M.E.S.); sonja.kanerva@helsinki.fi (S.K.); pia.vuorela@helsinki.fi (P.M.V.); 2 Pharmaceutical Sciences Laboratory, Faculty of Science and Engineering, Abo Akademi University, BioCity, Artillerigatan 6 A, FI-20520 Turku, Finland; suvi.manner@abo.fi

**Keywords:** quorum sensing, quorum sensing inhibition, *Chromobacterium violaceum*, screening, flavonoids, flavones, violacein quantification, assay optimization, anti-biofilm, microcolonies

## Abstract

Quorum sensing (QS) is the process by which bacteria produce and detect signal molecules to coordinate their collective behavior. This intercellular communication is a relevant target for anti-biofilm therapies. Here we have optimized a screening-applicable assay to search for new quorum sensing inhibitors from natural compound libraries. In this system, QS is correlated with the production of violacein, which is directly controlled by the LuxI/LuxR system in *Chromobacterium violaceum* ATCC 31532. The parallel use of *C. violaceum* Tn5-mutant CV026, which depends on auto-inducer addition, allows simultaneous discrimination of compounds that act as quenchers of the AHL signal (quorum quenchers). The incorporation of a redox stain into the platform allowed further distinction between QS inhibitors, quorum quenchers and antibacterial compounds. A pilot screening was performed with 465 natural and synthetic flavonoids. All the most active compounds were flavones and they displayed potencies (IC_50_) in the range of 3.69 to 23.35 μM. These leads were particularly promising as they inhibited the transition from microcolonies into mature biofilms from *Escherichia coli* and *Pseudomonas aeruginosa* strains. This approach can be very effective in identifying new antimicrobials posing lesser risks of resistance.

## 1. Introduction

Bacteria have developed several fine-tuned sensory systems to adequately and efficiently respond to environmental signals. The basic collective signaling system in biofilms is referred to as quorum sensing (QS), which is a process by which bacteria produce and detect signal molecules to coordinate their collective behavior in a cell-density dependent manner. This process was first discovered roughly 50 years ago, and a great deal of knowledge has now been accumulated. There are several QS systems, and they can be common or species specific; usually the signal molecules are kept species-specific to avoid cross-signaling in multi-species communities [1,2]. Many gram-negative organisms, including *Chromobacterium violaceum* and *Pseudomonas aeruginosa*, use acylhomoserine lactone (AHL)-type QS signals, which are synthetized by a LuxI-type synthase and are sensed by DNA-binding LuxR-type transcriptional activators [3,4]. Initiation of the QS signaling is dependent on population density to exceed a threshold; however, it has also been noticed that more actively communicating populations require a lower bacterial density [5].

Because of its relevance in cell-to-cell communication, QS is especially essential to biofilm populations. Biofilms are organized communities of bacteria embedded in a self-produced matrix, which are highly tolerant to most chemotherapeutic treatments as well as clearance by the host cell immune system [6]. This makes biofilms a challenging biomedical problem with yet unfound therapeutic solutions. It has been proposed that most microorganisms follow a programmed pathway for biofilm formation that involves a dynamic transition from single-state to microcolonies to the fully formed community. According to several lines of evidence, QS seems to play an essential role in the conversion of microcolonies into a mature biofilm, and thus QS inhibitors can potentially halt the transition into the most tolerant, recalcitrant stage. In addition, because QS is not essential for bacterial growth, QS inhibitors are expected to cause less selective pressure on bacteria than regular bactericidal treatments [7]. Thus, by targeting this communication system, it might be possible to find an effective new antimicrobial strategy without the immediate risk of developing resistance [8,9].

In particular, interference from the LuxI/LuxR QS system offers a unique path for developing novel anti-biofilm therapies because: (i) it increases the susceptibility of biofilms to antibiotics, thus reducing the dosage needed of these drugs to be effective on biofilms, (ii) it specifically affects only bacterial cells, and as mentioned earlier, (iii) it causes suppression of biofilm formation (in vitro & in vivo) without affecting bacterial growth, thus minimizing the risks of developing long-term resistance [10]. Consequently, this is driving a rapidly increasing interest in concerted translational projects to identify highly active, drug-like, non-toxic QS inhibitors as drug leads.

In this study we have used *Chromobacterium violaceum* as a model, which is a gram-negative, facultative anaerobe that is generally considered to be non-pathogenic for humans, but can cause severe septicemia, albeit in rare cases [11]. *C. violaceum* is an excellent reporter bacteria that produces a purple pigment known as violacein upon direct activation of LuxR-type proteins, such as CviR. This has made it widely attractive as reporter bacteria in QS studies. Here, we have optimized a screening assay to search for new QS inhibitors using the parent strain *C. violaceum* ATCC 31532 and its Tn5-mutant CV026 (NCTC 13278), which is deficient in AHL production and cannot produce violacein unless the autoinducer (C6-HSL) is added [3].

To be able to screen compounds with a higher throughput than in previously reported QS inhibition screening studies, we optimized both the growing conditions of the *C. violaceum* strains and the violacein quantification method. Previously, disc and well diffusion methods have been applied as qualitative screening methods for QS inhibitors, producing a yes/no output, often combined with a TLC overlay method [12,13,14]. Reports of quantification of violacein production have been earlier published in which drying (i.e., at 60 °C for 6–24 h) was used to sediment all biomass including the violacein, followed by dissolution in DMSO and photometric readout [15,16,17]. To facilitate the detection process, this method was further modified by introducing a biomass centrifugation step, allowing the violacein to be more efficiently dissolved in either *n*-butanol or DMSO for final photometric detection [13,14,18,19,20]. However, these above mentioned studies have been only conducted in low-throughput formats (i.e., tubes). In the present contribution, the originally proposed assay for violacein quantification by Choo et al. [19] was modified and optimized in a screening compatible setting, simultaneously using the *C. violaceum* strains ATCC 31532 and CV026. Because the CV026 strain depends on autoinducer addition for signal generation, potential quenchers of the QS signal (quorum quenchers) could be discriminated. Moreover, a counter-screening (viability-based) method was implemented, to distinguish between QS inhibitors and quorum quenchers from antibacterial hit compounds. With this strategy, it becomes feasible to quickly sort out chemical libraries for exhaustive identification of true virulence inhibitors.

Flavonoids are one of the most studied groups of natural products. They are widely spread in nature, for instance in various plant parts (i.e., leaves, flowers, seeds), and are present as essential part of our food [21]. Flavonoids are of great biomedical importance as they display activity against several biologically relevant targets. In the field of anti-infectives they have demonstrated antibacterial, antifungal and antiprotozoal activity [21,22,23]. Many recent studies have identified QS inhibitors from natural products and especially food-related sources [13,24,25,26]. Therefore, a library of flavonoids was deemed here as a plausible choice for a validatory QS inhibition screening campaign. The goal was to identify safe and novel virulence inhibitors, with no antibacterial activity, from naturally-derived flavonoids. 

## 2. Results and Discussion

### 2.1. Optimization of Growing Conditions and Violacein Production

*Chromobacterium violaceum* has been widely used for QS studies, but a wide range of growing conditions has been applied. To find the optimal conditions for both the growing and the violacein production, we performed a media screen where we used both recommended media TSB and LB (by the ATCC and NCTC from where the strains were purchased), together with different additives in several concentrations (Supplementary Table S1). Bacterial growth and violacein production were first visually inspected, and later on, when the optimal conditions had been chosen, statistical parameters were calculated and used for evaluation of the performance of the assay.

**Figure 1:**
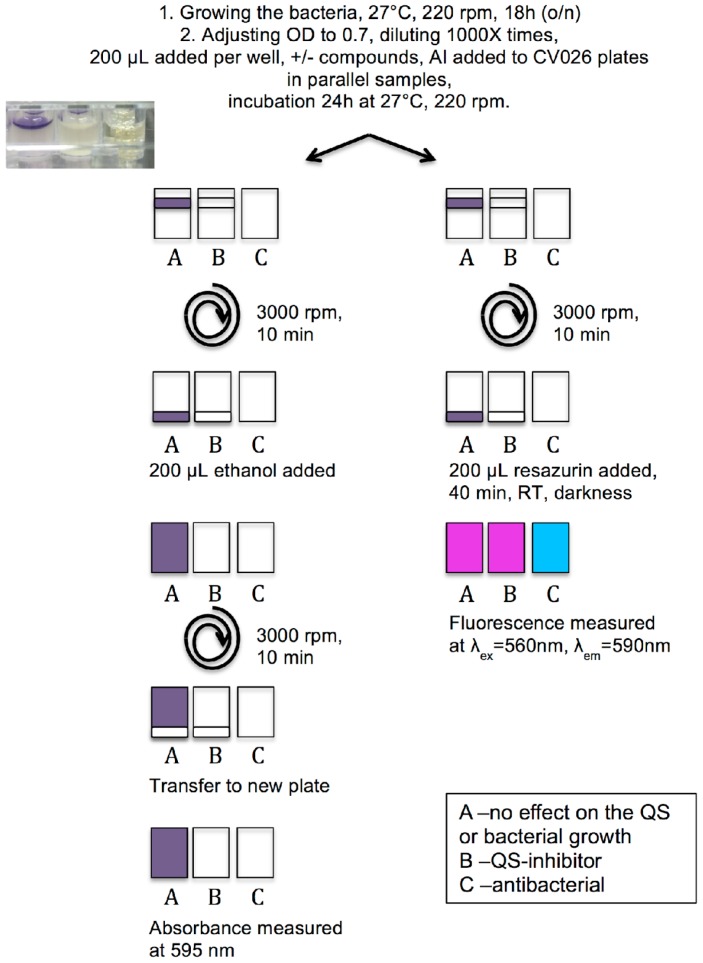
A schematic view of the workflow for the two assays of the platform. Parallel samples are used for violacein extraction (left pathway) and resazurin staining (right pathway). The rounded arrow indicates the centrifugation steps. The insert shows a photograph of the wells with samples of the different effects. From left to right: A represents a control well or a non-active compound, where the QS is active and violacein is produced. B represents an active QS inhibitor, where the violacein production is inhibited but the bacterial growth has not been altered (seen as turbid well). C represents the well of an antibacterial compound, where the bacteria have been completely killed and the medium is clear. AI addition stands for addition of autoinducer, 0.5 μM C_6_-HSL is used here.

The violacein quantification method was based on the protocol presented by Choo et al. [19], but modified to be applicable to microtiter well plates. The screening platform is schematically presented in Figure 1. The main challenge here was to quantify the violacein that is produced in the floating biofilm aggregates. Part of the biofilm population is attached to the bottom of the wells; however, the main part occurs as floating aggregates or complete biofilms on the surface (insert in Figure 1), which limits the accurate quantification of biofilms (mass, viability, violacein production). Therefore, by means of a first centrifugation step, all cells were sedimented, both colored and uncolored. The added ethanol dissolved the violacein but it was also found that scraping off the sedimented biofilms from the bottom significantly facilitated this dissolution process. Next, a second centrifugation step was performed to separate the cells from the extracellularly produced violacein and to avoid interference of cell turbidity in the absorbance measurement. Our choice of ethanol instead of DMSO as in Choo et al. [19] was based on practical reasons, as it makes the assay more applicable for screening by using a cheaper and less harmful solvent. Both solvents dissolved the violacein equally well (data not shown).

However, this violacein quantification procedure did not separate compounds with QSI activity from compounds that actually kill the bacteria, which ultimately would also interfere with the violacein production. For this purpose we applied resazurin staining of parallel samples, to access the viability of the treated bacteria. Resazurin is a well-known viability probe that is reduced from the blue, non-fluorescent form by active metabolism (NADH/H^+^ -> NAD^+^, H_2_O) to highly fluorescent, pink resorufin [27,28]. By combining these two assays we can detect QS inhibition activity and exclude bactericidal activity of the screened compounds. Additionally, using the CV026 mutant strain that has no AHL production of its own, it is possible to detect compounds that are able to degrade the signaling molecule before it reaches the bacteria and can be considered to be quorum quenchers. Therefore, the established platform can distinguish compounds with specific effects on QS from antibacterial compounds.

### 2.2. Screening for QS Inhibition Using a Flavonoid Collection

Quercetin at 400 μM was used as control of QS inhibition activity against both *C. violaceum* strains in our experimental conditions. This was a logical control here, since quercetin is a flavonol widely present in various foodstuff such as fruits, berries, tea and wine [29] and it has been shown to display QS inhibition activity against *C. violaceum* and *P. aeruginosa* [30,31]. Azithromycin was used as viability control and it prevented 100% of bacterial growth of both *C. violaceum* strains at 10 μM. The solvent of the flavonoid library, DMSO, was shown to enhance the violacein production. This DMSO effect on the violacein production of *C. violaceum* has been recently reported [18]. Thus, DMSO was added to the control wells together with the bacteria and the results are based on comparison to these DMSO-containing control wells. Statistical performance of the assay was monitored throughout the screening campaign and *Z′* of the screening plates (*n* = 14) were 0.47 ± 0.11 and 0.42 ± 0.15, for *C. violaceum* ATCC 31532 and *C. violaceum* CV026, respectively.

**Figure 2:**
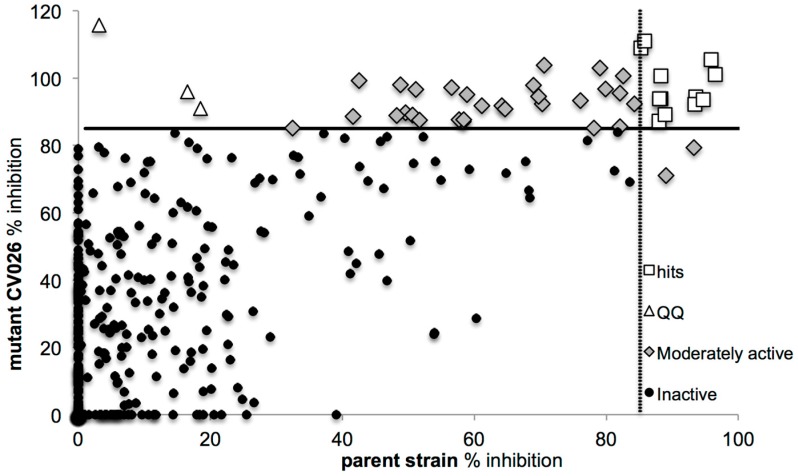
Screening of the flavonoids library (*n* = 465) for QS inhibition using the violacein extraction assay. The bactericidal compounds identified using the resazurin assay were excluded from the graph. White squares represent the hits that were active on both strains, grey squares represent the moderately active compounds while the white triangles represent the quorum quenchers (compounds active only on the mutant strain).

When screening the flavonoids collection (Figure 2 and Supplementary Table S2), a group of 70 compounds was classified as active, and that group included those compounds displaying over 85% inhibition on either both or one of the *C. violaceum* strains. From this group, 24 compounds were found to be antibacterial using the resazurin assay (showing more than 40% inhibition of bacterial viability) and were thus excluded from further testing (not shown in Figure 2). On the other hand, 31 of the non-bactericidal compounds were active only on one strain and therefore classified as moderately active. They are seen as grey squares in Figure 2, and they were not followed up. Three compounds (white triangles, Figure 2) did not show any activity (less than 20% inhibition) on the parent strain (ATCC 31532), but over 90% inhibition on the AHL-deficient CV026 mutant strain. For this strain the auto-inducer is added at the same time as the compounds, and thus it can be targeted and degraded before even reaching the bacterial cells, this way preventing the production of violacein. There are different mechanisms in which the AHL degradation can take place, but in all cases the process is known as quorum quenching [32]. Thus, these three compounds (F205, F317 and F325) were hypothesized to be quorum quenchers.

**Figure 3:**
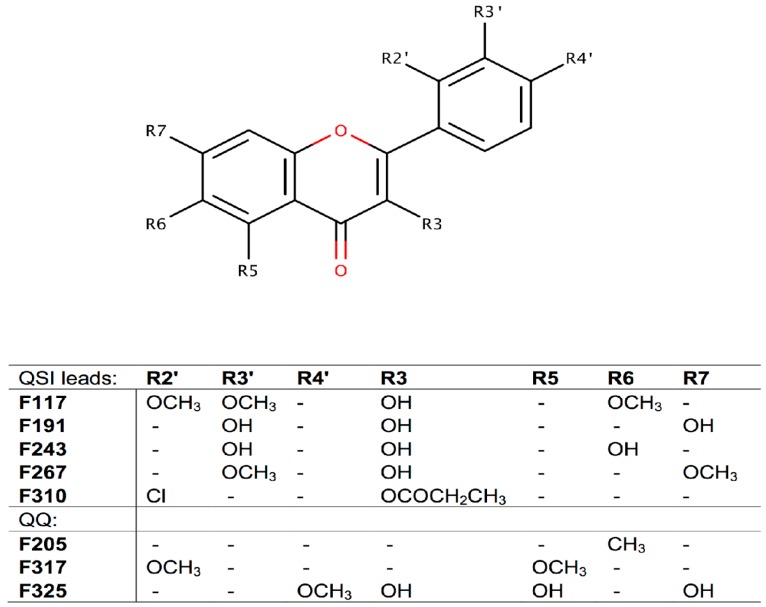
The flavone backbone and the varying substituents of the QS inhibition leads as well as the quorum quenchers. QSI indicates Quorum Sensing Inhibitors; QQ indicates Quorum Quenchers.

Of these three compounds with quorum quenching ability, F325 was identified as kaempferide, a flavonol found in the roots of the ginger family (Zingiberaceae). Kaempferide has been shown to lower the MIC of amoxicillin against amoxicillin resistant *E. coli* (AREC) when used in combination with this antibiotic [33]. These earlier findings together with our current results provide a strong support of the quorum quenching activity of kaempferide and its application in antimicrobial combination strategies. Compounds F205 and F317 were identified as 6-methylflavone and 2′,5-dimethoxyflavone, respectively, and to the best of our knowledge, these compounds have not been reported as quorum quenchers thus far. The structures of these three quorum quenchers are presented in Figure 3.

Finally, we identified 12 hits that were active QS inhibitors on both strains (white squares, Figure 2). The 12 hits were followed up by measuring their effects at a lower concentration (40 μM) using the parent *C. violaceum* strain. Those showing more than 90% inhibition at 40 μM were regarded as the lead compounds, and for those 5 active leads the concentration-response curves and potency (IC_50_) values were then obtained (Table 1).

All the five lead compounds were shown to display potency values (IC_50_ values) in the low micromolar range; the two most active leads, F267 and F243, had potencies of 3.69 and 8.73 µM, respectively. In addition, compound F191 that is structurally very closely related to compound F243, had an IC_50_ under 20 μM.

All the identified QS inhibitor leads, as well as the quorum quencher compounds (5 and 3 compounds, respectively), are flavones. The flavone backbone (2-phenyl-1,4-benzopyrone) and the substituents separately for each compound are presented in Figure 3. Four of the compounds (F117, F191, F243, and F267) share many substituents in their structures. It seems that small side groups, such as OH or OCH_3_ at the R2′- and R3′- positions, positively influence the QS inhibitor activity. The lead F310, which structurally differs the most from the others, is also the least active.

We further tested if these five QS inhibitor leads could affect the viability of biofilms formed by gram-negative bacteria: *E. coli* ATCC 10536 and ATCC 700928, as well as *P. aeruginosa* PAO1, ATCC 700829, ATCC 9027 and ATCC 15442 strains. Three out of the five leads (as well as the control compound, quercetin) had no inhibitory effect on the viability of the biofilms formed by these pathogenic strains (Supplementary Table S3). In contrast, F191 (3,7-dihydroxy-2-(3-hydroxyphenyl)-4H-chromen-4-one or 3,7,3′-trihydroxyflavone) displayed inhibitory activity (by 22%–50%) on the viability of biofilms formed by all strains. Additionally, F243 (3,6-dihydroxy-2-(3-hydroxyphenyl)-4H-chromen-4-one or 3,6,3′-trihydroxyflavone) showed some inhibitory effect on the viability of the *E. coli* biofilms (25%–41% inhibition), but no effect on the *P. aeruginosa* strains. Of note, from our previous research on this flavonoid collection [34], compound F191 was the only lead identified as moderately active against *S. aureus* biofilms (80% inhibition). Based on this, it could be argued that F191 has a broader antibacterial effect and it could also target virulence factors, such as QS in gram-negative bacteria. The high structural similarity to one of the most active leads, F243, suggests that this structural scaffold may play an important role in this antimicrobial effect.

**Figure 4:**
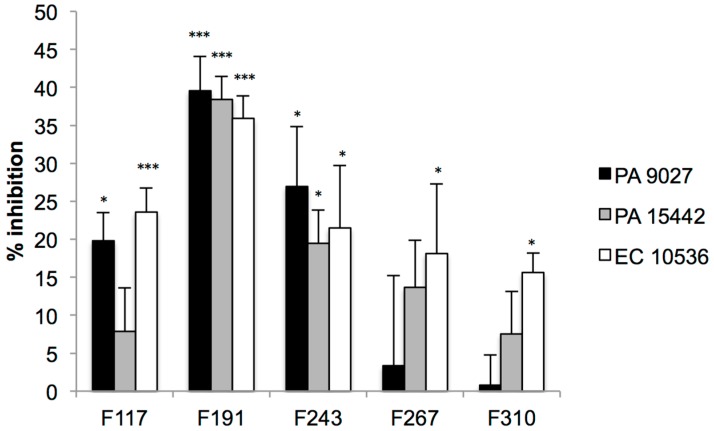
Activity of the QS inhibitors (leads, tested at 100 μM) on the transition between microcolonies and fully-formed biofilms of *E. coli* and *P. aeruginosa* strains (indicated as EC or PA strains in the legend). The figure contains results of crystal violet staining, shown as percent of inhibition when compared to untreated control samples. Error bars represent SEM (*n* = 6). *** *p* < 0.0001, * *p* < 0.05.

In the next stage, the ability of the five leads to target *P. aeruginosa* or *E. coli* microcolonies when they are transitioning to fully-formed biofilms was measured (Figure 4). All the QSI leads inhibited (to varying extents) the transition of pathogenic strains from microcolonies to mature biofilms. An inhibitory activity in this case is expected to be strongly connected to QSI. All of the initially identified hits also proved to be active in inhibiting a QS-driven process in one, two or three of the tested *E. coli* and *P. aeruginosa* strains. The inhibitory effects here were more significant for F191 and F243, but this is likely connected to the fact that they can also inhibit the viability of the biofilms that are formed under these conditions.

QS mechanisms have been studied extensively in *P. aeruginosa*, which uses several QS systems organized in a hierarchical manner. The LasI/LasR system, homologous to the LuxI/LuxR in *C. violaceum*, is in the top of the signaling hierarchy [35]. It has been argued that the LasI/R-system can be overridden by the RhlI/R-system if required [36]; however, the LasI/R-system is certainly one of the most important for a functional QS in *P. aeruginosa*. QS in *E. coli* has been studied in at least K12 and highly pathogenic EHEC strains and the mechanism seem to be a combination of different QS systems: *luxS*/AI-2, AI-3/epinephrine, indole signaling and the LuxR homolog SdiA-based system [37]. *E. coli* lacks the *luxI* gene that is responsible for the production of AHL molecules, but it has the LuxR homolog SdiA that monitors and binds AHL molecules from the environment [38]. Here, we may speculate that the LuxR homolog SdiA likely plays an important role as the leads disrupt the LuxI/LuxR system of *C. violaceum* and interfere with QS and biofilm formation in *E. coli*.

### 2.3. Conclusions

Here, we have optimized a workflow of two assays that can be used to screen for QS inhibition activity using chemical libraries. The developed concept allows us to distinguish QS inhibitors, quorum quenchers and antibacterial compounds, so it offers a potent tool for sorting out true virulence inhibitors. Both applied methods (violacein quantification for QS inhibition and resazurin for viability) use affordable reagents and materials, and the protocols are neither complicated nor time consuming. The exploratory screen conducted here allowed us to identify flavones, a very well known subclass of flavonoids, as QS inhibitors. Furthermore, we gathered evidence of several novel flavones with promising in vitro inhibitory activities on the biofilm lifecycle. These compounds provide a starting point for further investigations by our group. We expect that the developed assay platform will pave the way for a more systematic exploration of natural compound sources, which will allow the discovery of novel and potent virulence inhibitors.

## 3. Materials and Methods

### 3.1. Bacterial Strains

*Chromobacterium violaceum* (ATCC 31532), *Pseudomonas aeruginosa* ATCC 700829, ATCC 9027, ATCC 15442, *E. coli* strains ATCC 10536 and ATCC 700928 were all purchased from American type Culture Collection (ATCC; Wesel, Germany) and *C. violaceum* CV026 (NCTC 13278) from Public Health England′s National Culture of Type Collection (NCTC; Salisbury, UK). *Pseudomonas aeruginosa* PAO1 was obtained from University of Helsinki, HAMBI collection (http://www.helsinki.fi/hambi/). 

### 3.2. Optimization of Growing Conditions and Violacein Production by C. violaceum

Bacterial growing conditions and optimal violacein production were optimized by testing a range of growth media, media supplements and growing conditions, which are summarized in supplementary information (Supplementary Table S1). The final growing conditions used are presented here shortly. The liquid culture was made from 10 μL of a glycerol stock of the bacteria in 3 mL TSB (Sigma Aldrich, St. Louis, MO, USA) and incubated at 27 °C, 220 rpm aeration, for 18 h. The optical density was adjusted to 0.7 and this culture was diluted 1000 times to reach an initial bacterial concentration of 1 × 10^6^ CFU·mL^−1^. To improve the violacein production during biofilm formation LB medium (Serva Electrophoresis GmbH, Heidelberg, Germany) with additional yeast extract (0.1% *w/v*; Becton, Dickinson & Co., Le Pont de Claix, France) was used. For biofilm formation, 200 μL per well of bacteria solution was added onto sterile 96-micro well plates. C6-HSL (0.5 μM) was added to the CV026 cultures before plating. The plates were incubated at 27 °C, 200 rpm aeration, for 24 h. Serially diluted culture was routinely plated on agar for control of the initial bacteria concentration.

### 3.3. Protocol of Violacein Quantification by C. violaceum, Modified and Optimized for 96-Well Plates

The violacein quantification protocol by Choo et al. [19] was modified and optimized for 96-well plates. After the 24 h biofilm formation period the plates were centrifuged using a plate applicable rotor, in an Eppendorf 5810 R centrifuge (Thermo Fisher Scientific, Vantaa, Finland), at 3000 rpm, for 10 min, and the supernatant was removed using a multi channel pipette. The violacein was dissolved in 96% (*v/v*) ethanol, by gently scraping the bottoms of the wells using sterile plastic sticks. Then, the plates were centrifuged, as above, to sediment the bacterial cells. Finally, the colored solution in the supernatant (100 μL) was transferred to a new plate and the absorbance was measured at 595 nm, using a Varioskan LUX multimode plate reader (Thermo Scientific, Vantaa, Finland).

### 3.4. Measurement of Viability of C. violaceum Biofilms Using Resazurin Staining

Parallel biofilm samples (24 h grown) were used for viability staining with resazurin (Sigma Aldrich). The plates were centrifuged as described above (Section 3.3), the supernatant removed and resazurin stain added (200 μL per well, final resazurin concentration 20 μM), incubated for 30 min in darkness, 200 rpm aeration and fluorescence measured at λ_ex_ = 560 nm, λ_em_ = 590 nm, using Varioskan LUX.

### 3.5. QSI Screening of the Flavonoids Collection Using the Optimized C. violaceum Platform

For the pilot screening, a collection of 465 of flavonoids was obtained from TimTec (Newark, DE, USA). The library consists of a representative set of flavonoids from eight main classes: flavones, chalcones, flavonols, flavans, flavanones, isoflavonoids, neoflavonoids and dihydroflavonols. Compound identities were confirmed by the supplier, using Nuclear Magnetic Resonance spectroscopy (NMR) (300 MHz or higher) and Liquid Chromatography-Mass Spectrometry (LC/MS). Compound purities were measured using High-Performance Liquid Chromatography (HPLC) and ensured to be over 95%. The compounds were dissolved in dry DMSO and were tested at 400 μM. None of the compounds was found to interfere with the optical detection methods used here (described in Section 3.3 and Section 3.4). Quercetin, which is a flavonoid that has been shown to have QS inhibition activity, was used as positive control [30] while the antibiotic azithromycin was used as viability control. The effect of DMSO was evaluated by exposing the bacteria to 0.125%–5% DMSO for 24 h, in 96-well plates, using same conditions as in the screening.

### 3.6. Effect on the Viability of Biofilms Formed by Pathogenic Strains Using Resazurin Staining

For these studies *Escherichia coli*, ATCC 10536 and ATCC 700928, as well as *Pseudomonas aeruginosa* PAO1, ATCC 700829, ATCC 9027 and ATCC 15442 were utilized. The five lead compounds (at 400 μM) were added together with the bacteria into the wells and the effect on biofilm viability was evaluated using resazurin staining. This redox staining was performed as in Skogman et al. [39] with minor modifications. The planktonic solution was removed and the biofilms were washed once with sterile PBS, followed by addition of resazurin (20 μM) and incubation in the darkness, at room temperature for 45 min *(E. coli* biofilms) or 4 h (*P. aeruginosa* biofilms). Finally, the fluorescence was measured at λ_ex_ = 560 nm, λ_em_ = 590 nm, using Varioskan LUX.

### 3.7. Effect on the Transition from Microcolonies to Full-Formed Biofilms by Pathogenic Strains

The five QS inhibitors (lead compounds) were tested at 100 μM to evaluate the effect on the microcolonies and its transition to fully-formed biofilms. Strains used as in Section 3.6 were tested here (*Escherichia coli* ATCC 10536 as well as *Pseudomonas aeruginosa* ATCC 9027 and ATCC 15442). In this case, the bacteria were allowed to settle and form microcolonies for 2 h (37 °C, 220 rpm aeration). Then the medium and unattached bacteria was removed and replaced by fresh media containing the compounds for an additional 22 h incubation period (37 °C, 220 rpm aeration). Afterwards, plates were stained with crystal violet as in Skogman et al. [39] with minor modifications. The planktonic solution was removed and the biofilms were washed once with sterile PBS. The biomass was first fixed with methanol for 15 min at room temperature, then the wells were dried and stained with crystal violet (0.02%, *v/v*) for 5 min and washed twice with MQ-water before the remaining crystal violet was dissolved in 96% ethanol for 1 h and spectrophotometrically measured at 595 nm using Varioskan LUX.

### 3.8. Statistical Analysis

Potency (half inhibitory concentrations, IC_50_) calculations and Student′s *t*-test with Welch′s correction comparisons were conducted using GraphPad Prism 6 for Mac OS. Data were fitted using a sigmoidal curve with variable slope for the IC_50_ calculations. Statistical parameters (*Z′*, S/N, S/B, coefficient of variation, hit limits) for control of assay performance were calculated using Microsoft Excel for Mac throughout the optimization and screening process.

## Figures and Tables

**Table 1:** Potency values (IC_50_) of the five identified lead compounds.

	IC_50_ μM	95% Confidence Intervals	IC_50_ mg·L^−1^
F117	21.81	18.15 to 26.20	7.16
F191	19.83	17.73 to 22.18	5.36
F243	8.73	6.09 to 12.52	2.36
F267	3.69	2.92 to 4.67	1.10
F310	23.35	19.11 to 28.53	7.68
*Z′* for the follow up studies: 0.53 ± 0.2			

## Supplementary Materials

Supplementary materials can be accessed at: http://www.mdpi.com/1420-3049/21/9/1211/s1.
