# Supplementary material for: Flavones as Quorum Sensing Inhibitors Identified by a Newly Optimized Screening Platform Using Chromobacterium violaceum as Reporter Bacteria
Source: Molecules. 2016 Sep 10;21(9):1211. doi: 10.3390/molecules21091211 (PMC6273190; doi:10.3390/molecules21091211)
Supplement: Supplementary file 1 [file molecules-21-01211-s001.pdf]

Supplementary Materials: Flavones as Quorum Sensing Inhibitors Identified by a Newly Optimized Screening Platform Using *Chromobacterium violaceum* as Reporter Bacteria

Malena E. Skogman, Sonja Kanerva, Suvi Manner, Pia M. Vuorela and Adyary Fallarero

Table S1. Optimization conditions.

**ATCC 31532**			**Aerobic**		**Microaerophilic** §	
**Medium**	**Additive**	**Concentration**	**Growth**	**Violacein**	**Growth**	**Violacein**
TSB			+++	++	+++	++
TSB	glucose	1%	++	+	NT	NT
TSB	YE *	0.01%	+++	++	NT	NT
TSB	YE	0.10%	+++	+++	+++	++
TSB	YE	1%	+++	+++	NT	NT
TSB	gluc. + YE	1% + 1%	+++	+	NT	NT
TSB	EDTA	1%	-	-	-	-
LB			+++	++	+++	+
LB	glucose	1%	++	++	NT	NT
LB	YE	0.01%	+++	+++	NT	NT
LB	YE	0.10%	+++	+++	+++	+++
LB	YE	1%	+++	+	NT	NT
LB	gluc. + YE	1% + 1%	+++	+	NT	NT
LB	EDTA	1%	-	-	-	-
**CV026**						
TSB			+++	-	+++	-
TSB	AI **	0.1 μM	+++	+	NT	NT
TSB	AI	0.25 μM	+++	++	NT	NT
TSB	AI	0.5 μM	+++	+++	NT	NT
TSB	AI	1 μM	+++	+++	NT	NT
TSB-AI ***	YE	1%	+++	+++	NT	NT
TSB-AI	gluc. + YE	1% + 1%	+++	+	NT	NT
TSB-AI	EDTA	1%	-	-	-	-
LB-AI ***			+++	++	+++	+
LB-AI	glucose	1%	++	++	NT	NT
LB-AI	YE	0.01%	+++	+++	NT	NT
LB-AI	YE	0.10%	+++	+++	+++	++
LB-AI	YE	1%	+++	+	NT	NT
LB-AI	gluc. + YE	1% + 1%	+++	+	NT	NT
LB-AI	EDTA	1%	-	-	-	-

+++ :dark purple or very high turbidity 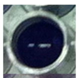

++ :light purple or less turbid 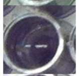

+ :very light purple 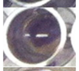

- :no color or no turbidity 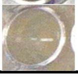

§ Microaerophilic: lower O_2 (3%) and richer CO_2 (17%) levels than the atmosphere; * YE -yeast extract; ** AI -autoinducer, C_6-HSL; *** TSB or LB medium containing 0.5 μM C_6-HSL.

Table S2. List of the library including data from the screening.

Cpd Code	TimTec Code	Parent Strain (ATCC 31532)			Mutant Strain CV026		
		Violacein Quantification (at 400 μM)	Follow up at 40 uM (Violacein)	Viability at 400 μM (Resazurin)	Violacein Quantification (at 400 μM)	Viability at 400 μM (Resazurin)	
		% Inhibition	% Inhibition	% Inhibition	% Inhibition	% Inhibition	Classification
1	ST001473	0.0			0.0		
2	ST001551	29.1			23.1		
3	ST001553	22.1			40.0		
4	ST002033	0.0			0.8		
5	ST002041	0.0			0.0		
6	ST002042	0.0			0.0		
7	ST002045	0.0			0.0		
8	ST002046	94.0		16.7	94.8	76.0	Bactericidal
9	ST002047	92.6		0.0	95.6	60.0	Bactericidal
10	ST002048	0.0			0.0		
11	ST002049	0.0			0.0		
12	ST002051	0.0			0.0		
13	ST002052	0.0			0.0		
14	ST002053	0.0			30.9		
15	ST002054	0.0			2.4		
16	ST002086	0.0			0.0		
17	ST002088	0.0			0.0		
18	ST002104	0.0			22.5		
19	ST004140	0.0			37.9		
20	ST004177	0.0			10.2		
21	ST004243	3.8			18.2		
22	ST005802	0.0			14.5		
23	ST007406	0.0			0.0		
24	ST008289	5.3			0.0		
25	ST008326	0.0			20.1		
26	ST008330	6.2			53.4		
27	ST008331	0.0			1.6		
28	ST014809	5.7			0.0		
29	ST019933	0.0			0.0		
30	ST019949	0.0			0.0		
31	ST020081	0.0			0.0		
32	ST020733	0.0			22.5		
33	ST020736	0.0			53.4		
34	ST021133	24.1			8.0		
35	ST021140	0.0			0.0		
36	ST023308	0.0			0.0		

37	ST024081	11.2			18.0		
38	ST024364	22.5			29.7		
39	ST024365	0.0			1.1		
40	ST024366	16.0			13.7		
41	ST024367	13.2			24.8		
42	ST024368	5.9			9.4		
43	ST024369	2.6			0.0		
44	ST024370	0.0			12.8		
45	ST024703	17.9			60.5		
46	ST024706	70.3		0.0	92.4	0.0	Moderately active
47	ST024709	0.0			0.0		
48	ST024713	0.0			56.5		
49	ST024793	0.0			53.1		
50	ST026594	0.0			0.0		
51	ST030560	33.6			71.4		
52	ST031278	0.0			7.1		
53	ST031314	0.0			3.1		
54	ST031315	0.0			0.0		
55	ST031318	43.9			69.4		
56	ST031321	7.0			0.0		
57	ST035556	7.6			0.0		
58	ST035557	0.0			0.0		
59	ST035691	0.0			0.0		
60	ST038026	10.9			40.2		
61	ST038325	59.2			72.8		
62	ST041961	34.9			59.1		
63	ST045882	93.6	2.2	0.0	94.5	0.0	HIT
64	ST046822	0.0			26.0		
65	ST048880	7.3			20.0		
66	ST049545	0.0			0.0		
67	ST051039	0.0			0.0		
68	ST051246	0.0			41.4		
69	ST055360	3.2			28.6		
70	ST055361	41.2			41.9		
71	ST055362	50.3			51.7		
72	ST055363	0.0			0.0		
73	ST055364	0.0			29.7		
74	ST055366	0.0			0.0		
75	ST055367	10.6			25.3		
76	ST055368	4.9			36.9		
77	ST055369	0.0			44.4		
78	ST055624	17.1			36.4		
79	ST055650	79.8		1.1	97.5	89.3	Bactericidal

80	ST055657	0.0			11.4		
81	ST055658	49.6		0.0	89.7	0.0	Moderately active
82	ST055981	88.3	80.7	0.0	93.9	0.0	HIT
83	ST055982	83.6			69.1		
84	ST055983	0.0			48.9		
85	ST055984	0.0			31.7		
86	ST055986	0.0			36.9		
87	ST055987	46.8			82.5		
88	ST055988	0.0			14.2		
89	ST055989	11.8			0.0		
90	ST055991	89.0		0.0	85.8	93.4	Bactericidal
91	ST055992	60.3			28.6		
92	ST055993	81.2			72.5		
93	ST055994	0.0			5.0		
94	ST055995	0.0			0.0		
95	ST055996	0.0			0.0		
96	ST055997	0.0			47.5		
97	ST055998	0.0			40.3		
98	ST055999	0.0			16.7		
99	ST056000	0.0			36.4		
100	ST056001	0.0			7.5		
101	ST056002	11.1			50.6		
102	ST056003	40.4			82.1		
103	ST056004	8.7			3.4		
104	ST056007	0.0			0.0		
105	ST056008	45.5			47.6		
106	ST056009	0.2			41.6		
107	ST056010	14.4			59.9		
108	ST056012	98.5		100.9	97.9	95.6	Bactericidal
109	ST056013	27.6			54.4		
110	ST056014	58.3		19.1	87.1	0.0	Moderately active
111	ST056194	54.9			69.7		
112	ST056228	0.0			46.4		
113	ST056229	0.0			17.5		
114	ST056246	54.1			75.3		
115	ST056247	42.6			73.8		
116	ST056248	64.8			71.7		
**117**	ST056249	**88.0**	**90.6**	0.0	**87.3**	0.0	**HIT**
118	ST056250	46.7			39.8		
119	ST056251	22.7			48.9		
120	ST056252	19.6			55.9		
121	ST056253	42.1			44.9		
122	ST056254	0.0			1.9		

123	ST056255	0.0			29.3		
124	ST056256	6.9			52.9		
125	ST056258	19.5			76.0		
126	ST056259	41.6		0.0	88.6	0.0	Moderately active
127	ST056288	59.1		82.3	100.1	98.0	Bactericidal
128	ST056301	0.0			2.2		
129	ST056352	19.0			38.3		
130	ST057152	97.1		24.9	99.6	99.2	Bactericidal
131	ST057176	25.5			0.0		
132	ST057233	78.1		0.0	85.2	0.0	Moderately active
133	ST057235	84.2		0.0	92.3	47.7	Moderately active
134	ST057236	18.7			34.9		
135	ST057247	0.0			0.0		
136	ST057268	83.7		17.1	99.5	99.4	Bactericidal
137	ST057370	0.0			13.7		
138	ST057371	0.0			0.0		
139	ST057372	0.0			7.4		
140	ST057515	21.7			0.0		
141	ST057541	0.3			0.0		
142	ST057580	97.1		0.0	90.5	98.6	Bactericidal
143	ST057630	4.9			0.0		
144	ST057634	0.0			0.0		
145	ST057635	11.5			0.0		
146	ST057636	54.0			24.3		
147	ST057637	0.0			0.0		
148	ST057638	0.6			38.5		
149	ST057639	8.1			0.0		
150	ST057640	69.7		30.2	94.6	12.8	Moderately active
151	ST057641	0.0			43.4		
152	ST057643	0.0			0.0		
153	ST057644	0.0			10.5		
154	ST057645	0.0			14.6		
155	ST057646	0.0			34.4		
156	ST057647	56.5		43.5	97.2	50.5	Moderately active
157	ST057648	0.0			38.9		
158	ST057649	42.6		0.0	99.3	0.0	Moderately active
159	ST057656	40.4		16.6	99.9	94.8	Bactericidal
160	ST057709	40.9			48.5		
161	ST057710	88.1	53.7	30.4	93.8	0.0	HIT
162	ST057711	67.7			75.2		
163	ST057729	0.0			65.1		
164	ST057770	3.6			29.2		
165	ST058412	7.6			41.5		

166	ST058414	10.5			33.7		
167	ST058433	9.2			56.1		
168	ST058434	14.1			41.1		
169	ST058442	4.3			31.8		
170	ST058451	7.3			23.9		
171	ST058458	23.3			76.3		
172	ST058459	18.4			43.7		
173	ST059080	0.0		24.6	92.4	92.1	Bactericidal
174	ST059081	14.2			50.8		
175	ST059082	0.0			25.1		
176	ST059299	69.0		NT	97.9	NT	Moderately active
177	ST059300	14.7			19.1		
178	ST059590	9.6			0.0		
179	ST059616	3.6			44.2		
180	ST059618	64.2		0.0	91.8	0.0	Moderately active
181	ST059619	85.2	75.6	0.0	108.9	0.0	HIT
182	ST059620	1.1			34.0		
183	ST059621	57.7		48.7	87.6	7.6	Moderately active
184	ST059622	91.8		43.3	93.5	46.2	Bactericidal
185	ST059828	0.0			0.0		
186	ST059837	85.8	9.6	3.6	111.1	0.0	HIT
187	ST059919	0.0			0.0		
188	ST059922	8.0			36.1		
189	ST059923	22.6			20.8		
190	ST059924	5.9			0.0		
**191**	ST059925	**93.5**	**93.9**	0.0	**92.3**	0.0	**HIT**
192	ST060160	14.4			6.3		
193	ST060287	18.8			19.5		
194	ST060837	0.0			0.0		
195	ST065428	16.8			39.5		
196	ST066889	32.6			77.0		
197	ST066904	16.8			80.8		
198	ST066973	3.3			0.0		
199	ST069293	70.6		0.0	103.8	0.0	Moderately active
200	ST069294	82.1		21.1	85.6	0.0	Moderately active
201	ST069305	13.1			36.1		
202	ST069306	0.0			0.0		
203	ST069307	79.1		0.0	103.0	0.0	Moderately active
204	ST069324	16.6			40.8		
205	ST069348	3.2		45.8	115.8	0.0	QQ
206	ST069360	46.2			67.2		
207	ST069833	20.2			7.6		
208	ST070122	0.0			0.0		

209	ST070123	10.6			0.0		
210	ST070175	20.2			13.7		
211	ST070176	11.5			0.0		
212	ST070177	7.0			6.7		
213	ST070178	11.8			52.6		
214	ST070182	0.0			9.8		
215	ST070203	0.0			17.9		
216	ST070242	12.7			34.5		
217	ST070262	10.5			75.1		
218	ST070287	0.0			0.0		
219	ST070634	1.6			0.0		
220	ST070865	18.0			79.0		
221	ST070866	51.1		7.6	96.7	0.0	Moderately active
222	ST070867	0.0			54.3		
223	ST070868	68.2			66.7		
224	ST070869	52.3			82.6		
225	ST070967	0.8			43.3		
226	ST072170	0.0			0.0		
227	ST072640	100.2		97.8	98.5	96.3	Bactericidal
228	ST072642	0.0			72.9		
229	ST072691	0.0			0.5		
230	ST072734	0.0			0.0		
231	ST074453	0.0			45.1		
232	ST074454	0.0			46.4		
233	ST074455	50.8			74.6		
234	ST074456	33.3			76.4		
235	ST074488	26.8			68.9		
236	ST074501	0.0			57.0		
237	ST074502	0.0			0.0		
238	ST074528	0.0			12.8		
239	ST074529	2.3			65.8		
240	ST074531	0.0			12.4		
241	ST074554	0.0			54.3		
242	ST074555	6.3			54.3		
**243**	ST074562	**96.5**	**96.9**	0.0	**101.1**	0.0	**HIT**
244	ST074635	0.0			0.0		
245	ST075446	82.6		0.0	100.7	0.0	Moderately active
246	ST075648	58.9		52.4	95.1	18.7	Moderately active
247	ST076514	0.0			0.0		
248	ST076515	0.0			9.5		
249	ST076521	17.0			15.9		
250	ST076522	0.0			0.0		
251	ST076527	6.9			2.8		

252	ST076528	32.5		16.0	85.2	0.0	Moderately active
253	ST076530	6.0			67.8		
254	ST076533	3.2			36.9		
255	ST076534	8.6			33.3		
256	ST076546	0.0			63.0		
257	ST076550	7.1			76.1		
258	ST077089	14.7			83.6		
259	ST077108	50.6		0.0	89.1	0.0	Moderately active
260	ST077124	64.6		NT	90.8	NT	Moderately active
261	ST077133	17.9			0.0		
262	ST077139	0.0			0.0		
263	ST078083	15.6			63.0		
264	ST078115	6.6			19.8		
265	ST078351	0.0			0.0		
266	ST078865	88.9	42.2	0.0	89.2	0.0	HIT
**267**	ST078866	**94.7**	**93.5**	0.0	**93.6**	0.0	**HIT**
268	ST079062	11.6			64.2		
269	ST079153	0.0			78.9		
270	ST079165	0.0			0.8		
271	ST079166	7.7			3.2		
272	ST079167	0.0			0.0		
273	ST079544	0.3			0.0		
274	ST079545	4.0			18.2		
275	ST079546	20.5			0.0		
276	ST079547	0.0			0.0		
277	ST079548	0.9			0.0		
278	ST079564	0.0			46.4		
279	ST079566	0.0			30.6		
280	ST079948	0.0			12.9		
281	ST079949	3.1			18.8		
282	ST079950	5.0			0.0		
283	ST079951	26.4			30.6		
284	ST079954	5.7			25.7		
285	ST079961	0.4			20.8		
286	ST079964	0.9			42.5		
287	ST080006	4.5			25.2		
288	ST080007	18.9			0.0		
289	ST080024	22.8			29.2		
290	ST080274	5.9			50.4		
291	ST080500	0.0			0.0		
292	ST080501	36.7			64.7		
293	ST080512	0.0			27.7		
294	ST080603	3.1			79.5		

295	ST080606	89.1		0.0	71.1	0.0	Moderately active
296	ST081006	0.0			6.7		
297	ST081327	0.0			0.0		
298	ST081356	98.4		90.6	99.8	94.0	Bactericidal
299	ST081387	0.0			37.6		
300	ST081388	4.1			0.0		
301	ST081389	0.0			17.8		
302	ST081567	0.0			53.4		
303	ST081587	5.4			26.7		
304	ST081588	0.0			0.0		
305	ST081598	0.0			0.0		
306	ST081626	10.1			65.7		
307	ST082229	76.1		0.0	93.3	0.0	Moderately active
308	ST083078	0.0			24.7		
309	ST083089	10.0			39.9		
**310**	ST083092	**95.9**	**92.1**	0.0	**105.5**	0.0	**HIT**
311	ST083122	0.0			10.9		
312	ST083129	23.1			16.3		
313	ST083130	1.8			48.6		
314	ST083652	9.1			40.8		
315	ST083653	22.3			45.3		
316	ST083654	7.9			69.0		
317	ST083655	16.5		0.0	96.0	0.0	QQ
318	ST083656	3.0			47.8		
319	ST083657	3.1			15.0		
320	ST083658	19.4			25.0		
321	ST083683	20.3			55.7		
322	ST085121	18.3			0.0		
323	ST085666	6.0			0.0		
324	ST085667	6.4			0.0		
325	ST085668	18.5		0.0	91.0	0.0	QQ
326	ST085669	12.3			30.0		
327	ST085670	29.4			69.8		
328	ST085671	61.1		0.0	91.8	0.0	Moderately active
329	ST085672	79.9		0.0	96.8	0.0	Moderately active
330	ST085906	23.5			44.5		
331	ST086116	68.4			64.4		
332	ST086243	93.3		0.0	79.4	0.0	Moderately active
333	ST086510	0.0			0.0		
334	ST086511	82.1		0.0	95.5	0.0	Moderately active
335	ST086622	58.4		0.0	87.7	0.0	Moderately active
336	ST088823	0.4			0.0		
337	ST090287	4.3			0.0		

338	ST092286	0.0		0.0		
339	ST092287	4.2		0.0		
340	ST092291	37.2		83.5		
341	ST092293	38.3	0.0	95.2	100.6	Bactericidal
342	ST092296	48.8	0.0	98.1	16.5	Moderately active
343	ST092297	0.0		5.0		
344	ST092710	0.0		0.0		
345	ST092742	0.0		0.0		
346	ST092763	0.0		22.4		
347	ST092764	0.0		36.1		
348	ST093690	0.0		67.8		
349	ST094800	0.0		0.0		
350	ST094806	0.0		0.0		
351	ST094856	3.4		0.0		
352	ST094874	0.0		44.4		
353	ST094877	0.0		27.7		
354	ST094880	0.0		54.2		
355	ST094881	1.4		11.1		
356	ST094882	0.0		12.6		
357	ST094883	0.0		11.1		
358	ST094884	0.0		0.0		
359	ST098835	51.7	0.0	87.5	0.0	Moderately active
360	ST097864	0.0		69.3		
361	ST097621	26.6		3.5		
362	ST095190	0.2		20.2		
363	ST095191	0.0		0.0		
364	ST095192	0.0		0.0		
365	ST095193	0.0		38.3		
366	ST095194	0.0		61.0		
367	ST095195	1.2		56.5		
368	ST095196	0.0		67.8		
369	ST095197	0.0		8.8		
370	ST095198	0.0		0.0		
371	ST095199	0.0		0.0		
372	ST095200	0.0		2.8		
373	ST095201	0.0		13.8		
374	ST095202	27.5		70.2		
375	ST095227	0.0		35.5		
376	ST095411	99.1	85.9	99.7	73.1	Bactericidal
377	ST095412	77.1		81.4		
378	ST095413	3.6	96.1	95.2	84.9	Bactericidal
379	ST095414	37.3	0.0	89.2	91.6	Bactericidal
380	ST095416	17.9		46.6		

381	ST095417	96.4		88.3	93.9	53.3	Bactericidal
382	ST095418	93.0		97.8	89.8	97.3	Bactericidal
383	ST095419	0.0			0.0		
384	ST097570	0.0			0.0		
385	ST095424	0.0			3.8		
386	ST095787	94.1		91.1	93.7	82.7	Bactericidal
387	ST095788	68.2		82.1	96.1	94.1	Bactericidal
388	ST095795	0.0			0.0		
389	ST095849	45.8			81.2		
390	ST095850	0.0			0.0		
391	ST095851	0.0			0.0		
392	ST095852	0.0			0.0		
393	ST096006	0.0			55.3		
394	ST096007	0.0			11.9		
395	ST096010	0.0			32.4		
396	ST096990	0.0			6.3		
397	ST097090	0.0			0.0		
398	ST097091	0.0			0.0		
399	ST097092	16.3			41.1		
400	ST097093	21.4			10.0		
401	ST097094	4.0			8.2		
402	ST097097	0.0			31.8		
403	ST097099	0.0			0.0		
404	ST097101	0.0			5.7		
405	ST097107	10.9			0.0		
406	ST097108	11.1			27.4		
407	ST097110	0.0			0.0		
408	ST097111	0.0			0.0		
409	ST097117	0.0			10.0		
410	ST097121	0.0			28.7		
411	ST097125	10.9			49.1		
412	ST097570	0.0			0.0		
413	ST097864	14.4			31.9		
414	ST098360	20.8		0.0	100.3	54.1	Bactericidal
415	ST098799	0.0			0.0		
416	ST098835	81.7			83.9		
417	ST031410	53.9			23.9		
418	ST032792	4.2			16.6		
419	ST055359	0.0			34.2		
420	ST055365	4.7			52.6		
421	ST055990	3.3			0.0		
422	ST056006	0.0			51.8		
423	ST056011	48.2	19.3	47.0	88.9	0.0	Moderately active

424	ST097095	7.7			12.4		
425	ST097096	0.0			22.8		
426	ST097100	6.0			9.8		
427	ST097104	6.5			17.4		
428	ST097105	2.5			27.0		
429	ST097106	4.7			24.3		
430	ST097122	0.0			33.3		
431	ST097123	0.0			37.6		
432	ST097132	0.0			27.3		
433	ST097133	0.0			20.8		
434	ST097134	0.0			25.3		
435	ST097142	6.0			54.4		
436	ST097143	6.6			26.6		
437	ST097144	0.0			24.1		
438	ST097145	0.0			34.8		
439	ST097428	88.3	43.0	0.0	100.7	4.2	HIT
440	ST097701	0.4			36.6		
441	ST098942	0.0			76.9		
442	ST098943	24.8			4.5		
443	ST098944	0.0			0.0		
444	ST098945	0.0			12.8		
445	ST098946	28.2			54.1		
446	ST098947	5.7			40.3		
447	ST098948	3.9			25.6		
448	ST098949	0.0			33.3		
449	ST098950	1.0			37.6		
450	ST098951	11.2			27.3		
451	ST098952	17.1			20.8		
452	ST098953	14.4			25.3		
453	ST098954	39.1			54.4		
454	ST098955	16.6			26.6		
455	ST098956	0.0			24.1		
456	ST098957	18.9			34.8		
457	ST098958	10.0		31.9	100.7	71.8	Bactericidal
458	ST098959	3.9			36.6		
459	ST098960	1.5			76.9		
460	ST098961	11.8			4.5		
461	ST098962	9.6			0.0		
462	ST098963	5.6			12.8		
463	ST098976	6.5			54.1		
464	ST098978	10.9			40.3		
465	ST098979	19.1			25.6		
QUE	Quercetin	70.5		0.0	93.4	0.0	Control

HIT (*n* = 12): active on both strains (>85% inhibition), not bactericidal; **LEAD** (*n* = 5): the most active compounds, (IC_{50}s 3.69–23.35 μM); **QQ** (*n* = 3): active on the mutant CV026 strain (>85% inhibition), but <20% inhibition on the parent strain, Quorum Quenchers; **Moderately active** (*n* = 31): active on one strain (>85% inhibition), 20%–84% inhibition on the other strain; **Bactericidal** (*n* = 24): bactericidal to either one or both of the strains.

Statistical performance of the assay during the screening:

	***C. violaceum* ATCC 31532**		***C. violaceum* CV026**	
	**mean**	**SD**	**mean**	**SD**
S/N	6.29	1.54	5.86	1.74
S/B	8.29	1.17	4.70	1.54
Z'	0.47	0.11	0.42	0.15

Table S3. Effect on biofilms viability measured for all compounds against all *P. aeruginosa* and *E. coli* strains.

		Viability at 400 μM (Resazurin)		
		**% Inhibition**	**±**	**SEM**
EC10536	F117	10.48	±	4.05
	F191	29.25	±	2.37
	F243	24.67	±	0.33
	F267	−0.13	±	3.39
	F310	0.02	±	3.03
	QUE	−6.66	±	2.65
EC700928	F117	12.73	±	4.80
	F191	35.03	±	0.07
	F243	40.89	±	0.45
	F267	−26.54	±	2.22
	F310	−1.98	±	2.41
	QUE	1.54	±	2.66
PA9027	F117	−32.42	±	14.71
	F191	49.45	±	11.61
	F243	−1.71	±	61.04
	F267	−37.64	±	10.70
	F310	−27.36	±	30.76
	QUE	−3.99	±	27.89
PA15442	F117	−24.15	±	6.07
	F191	22.19	±	24.13
	F243	−12.62	±	21.16
	F267	−23.02	±	3.18
	F310	−19.37	±	10.95
	QUE	−23.76	±	4.03
PAO1	F117	14.50	±	3.19
	F191	52.83	±	33.62
	F243	4.65	±	6.66
	F267	−6.31	±	9.28
	F310	−20.44	±	1.14
	QUE	8.49	±	1.07
PA700829	F117	6.68	±	8.04
	F191	30.36	±	1.43
	F243	−6.65	±	0.42
	F267	−10.49	±	9.56
	F310	−26.18	±	7.96
	QUE	−4.97	±	7.97

QUE-quercetin
